# Orphaned female elephant social bonds reflect lack of access to mature adults

**DOI:** 10.1038/s41598-017-14712-2

**Published:** 2017-10-31

**Authors:** Shifra Z. Goldenberg, George Wittemyer

**Affiliations:** 1 Department of Fish, Wildlife, and Conservation Biology, Fort Collins, CO 80523 USA; 2 Save the Elephants, Nairobi, 00200 Kenya

## Abstract

Compensatory social behavior in nonhuman animals following maternal loss has been documented, but understanding of how orphans allocate bonding to reconstruct their social networks is limited. Successful social integration may be critical to survival and reproduction for highly social species and, therefore, may be tied to population persistence. We examined the social partners involved in affiliative interactions of female orphans and non-orphans in an elephant population in Samburu, northern Kenya that experienced heightened adult mortality driven by drought and intense ivory poaching. We contrasted partners across different competitive contexts to gain insight to the influence of resource availability on social interactions. Though the number of partners did not differ between orphans and non-orphans, their types of social partners did. Orphans interacted with sisters and matriarchs less while feeding than did non-orphans, but otherwise their affiliates were similar. While resting under spatially concentrated shade, orphans had markedly less access to mature adults but affiliated instead with sisters, bulls, and age mates. Orphan propensity to strengthen bonds with non-dominant animals appears to offer routes to social integration following maternal loss, but lack of interaction with adult females suggests orphans may experience decreased resource access and associated fitness costs in this matriarchal society.

## Introduction

Strong mother-offspring bonds are widespread in philopatric species^1^. The importance of these bonds is demonstrated by the adverse consequences documented for orphans across species, including lower life expectancy^2,3^, decreased physical condition^4,5^, and stunted vocal behavior^6^. Social processes may be especially predictive of the consequences of orphaning. For example, offspring survival in baboons^7^ and horses^8^ is tied to the social relationships of mothers. The absence of those social relationships may therefore be expected to have detrimental effects on orphans, especially for species highly dependent on social bonds^2,9,10^. Remaining socially integrated presents a challenge for orphans given the loss of their mothers’ social influence. However, compensatory behavior has been documented in some species^11–14^, and access to important social partners is known to increase survival^15,16^. Orphans may therefore strive to mitigate the impacts of maternal loss through compensatory social behavior. Though compensatory bonding is not well studied in nonhuman animals, it may be an important avenue by which sociality influences fitness.

Social behavioral comparisons between orphans and non-orphans provide a powerful framework to explore compensatory bonding. Because the number of strong relationships individuals can maintain is limited, animals should bond with those that offer the greatest social benefit. In female baboons, preferences follow a hierarchy of availability of relatives: in the absence of mothers, baboons bond strongly with maternal kin, and in the absence of maternal kin they bond with paternal kin or nonrelatives^12^. Baboon society is highly nepotistic^17^, and the prevalence of these patterns across taxa with different social structures is unknown. In species disproportionately reliant on older individuals^18,19^, bonding with older conspecifics following maternal loss may be expected to take precedence over bonding with relatives.

Female African savannah elephants (*Loxodonta africana*) form matriarchal societies in which daughters remain with their mothers and other female relatives in multi-generational groups for life^20,21^. The disproportionate poaching of older elephants for their larger ivory^22,23^ removes critical social partners that offer ecological knowledge and resource access^19,24,25^. Aberrant behavior has been recorded in male elephant orphans that experienced impoverished social environments^10^. Recovery of elephant populations may in part depend on the ability of orphans to remain socially integrated following maternal loss. Because of their decades-long reliance on their mothers and the relationship of maternal loss to the current ivory poaching crisis^26,27^, elephants provide a highly relevant wild system in which to investigate orphan bonding patterns.

Elephants’ social patterns are structured by relatedness, as in other species^1,21,28^, but studies of disrupted populations suggest bonds with nonrelatives may replace those with relatives^29–32^. The weak influence of nepotism in elephants^33,34^ also indicates a reduced reliance on kin relative to other species^17^. In this study, we extend previous work on partner preference^12^ to a different social system to broaden understanding of the process of orphan social integration. We followed the social behavior of orphan and non-orphan female elephants in the Samburu population in northern Kenya following drought and during a persistent period of poaching^22,26^, testing the prediction that orphans shift allocation of bonding effort to maternal relatives. Because resource access structures social interaction, we separated behaviors during foraging of generally widely distributed resources and resting in spatially concentrated shade. We expected orphans to interact less with dominant individuals while resting when competition is greater. We discuss the implications of our results for long term orphan social integration and elephant population recovery.

## Results

Number of affiliative partners per time followed did not significantly differ between orphans and non-orphans at the α = 0.05 level (Kruskal-Wallis Rank Sum Test: Feeding: χ^2^ = 0.017, p = 0.897; Resting: χ^2^ = 0.004, p = 0.949). However, we found a significantly lower age difference between orphans and their adult/older female social partners (excluding bulls) while resting (Kruskal-Wallis Rank Sum Test: χ^2^ = 6.259, p = 0.012) but not while feeding (Kruskal-Wallis Rank Sum Test: χ^2^ = 1.855, p = 0.173) relative to that of non-orphans. Related to the generally stronger interactions with bulls by orphans relative to non-orphans, we did not find significant differences in the age differences between the two across all social partners (Feeding: χ^2^ = 1.603, p = 0.206; Resting: χ^2^ = 0.483, p = 0.487).

**Figure 1:**
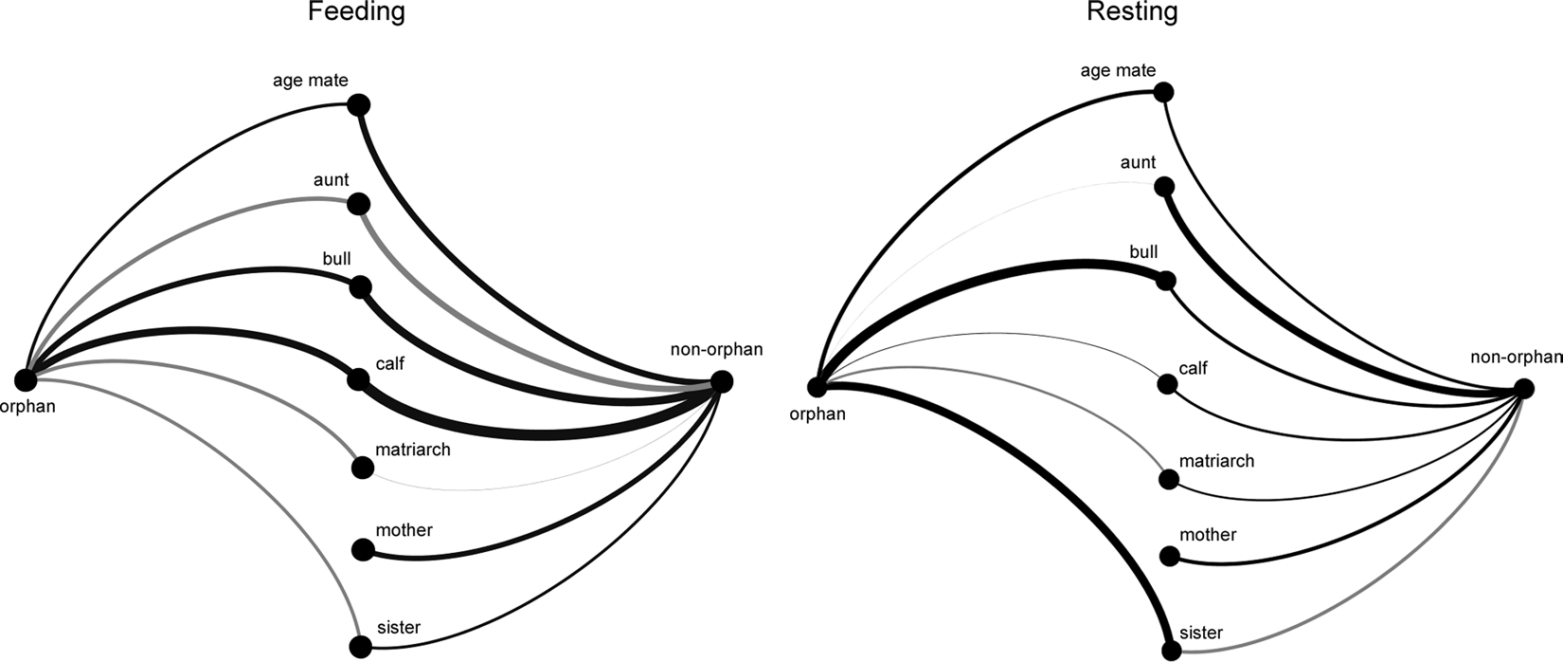
Regression median estimates associated with social partner category affiliation. Thicker lines represent stronger probabilities. Black and gray lines represent positive and negative coefficients, respectively, such that thick black lines indicate high probability of affiliation whereas thick gray lines indicate high probability of not affiliating (avoidance).

While feeding, both orphans and non-orphans preferentially interacted with calves, age mates, and bulls (Tables 1 and 2; Fig. 1). Non-orphans were more likely to interact with sisters relative to orphans, and demonstrated preference for their mothers. Aunts were unlikely feeding affiliation partners for both orphans and non-orphans, and orphans were unlikely to affiliate with matriarchs when feeding. Younger orphans demonstrated more feeding affiliation, but orphans that lost their mothers at an older age had more affiliative interactions while feeding (Table 1).

**Table 1:** Median (95% credible interval) posterior distribution estimates of regression coefficients. Columns distinguish covariates included in models and rows distinguish models.

	Age	Age orphaned	Age mate	Aunt	Bull	Calf	Matriarch	Mother	Sister
Orphans (feeding)	−0.446 (−0.735–−0.164)	0.154 (−0.135–0.438)	0.390 (−0.009–0.794)	−0.521 (−1.568–0.641)	0.702 (0.291–1.115)	0.900 (0.559–1.241)	−0.451 (−1.069–0.185)		−0.404 (−1.009–0.230)
Non-orphans (feeding)	0.068 (−0.142–0.255)		0.761 (0.281–1.256)	−0.730 (−1.764–0.361)	0.963 (0.437–1.497)	1.298 (0.908–1.690)	−0.036 (−1.093–1.047)	0.631 (0.064–1.239)	0.345 (−0.214–0.945)
Orphans (resting)	−0.054 (−0.452–0.370)	−0.238 (−0.672–0.172)	0.533 (−0.033–1.124)	−0.014 (−1.246–1.460)	1.210 (0.404–2.062)	0.089 (−0.478–0.675)	−0.299 (−1.077–0.552)		1.023 (0.386–1.704)
Non-orphans (resting)	0.028 (−0.203–0.272)		0.376 (−0.286–1.073)	0.935 (−0.694–3.050)	0.435 (−0.382–1.333)	0.326 (−0.290–0.951)	0.252 (−1.176–2.053)	0.477 (−0.222–1.221)	−0.423 (−1.246–0.534)

**Table 2:** Median (IQR) interaction rates with partner categories for orphans and non-orphans.

Activity	Feeding		Resting	
Partner category	Orphan	Non-orphan	Orphan	Non-orphan
Age mate	0.026 (0.011–0.067)	0.019 (0.006–0.080)	0.118 (0.059–0.408)	0.221 (0.093–0.470)
Aunt	0.009 (0.005–0.012)	0.007 (0.003–0.014)	0.123 (0.046–0.217)	0.707 (0.595–0.819)
Bull	0.033 (0.016–0.067)	0.030 (0.017–0.050)	0.367 (0.095–0.462)	0.095 (0.058–0.442)
Calf	0.031 (0.019–0.063)	0.027 (0.013–0.071)	0.144 (0.043–0.219)	0.217 (0.067–0.417)
Matriarch	0.012 (0.004–0.016)	0.014 (0.009–0.018)	0.175 (0.036–0.308)	0.333 (0.222–0.632)
Mother		0.017 (0.011–0.033)		0.192 (0.055–0.563)
Sister	0.015 (0.005–0.025)	0.032 (0.008–0.054)	0.300 (0.105–0.617)	0.100 (0.041–0.186)

Values are calculated as the number of affiliative interactions divided by the number of focal follow minutes during which both partners were observed in the same aggregation and therefore available to interact.

**Figure 2:**
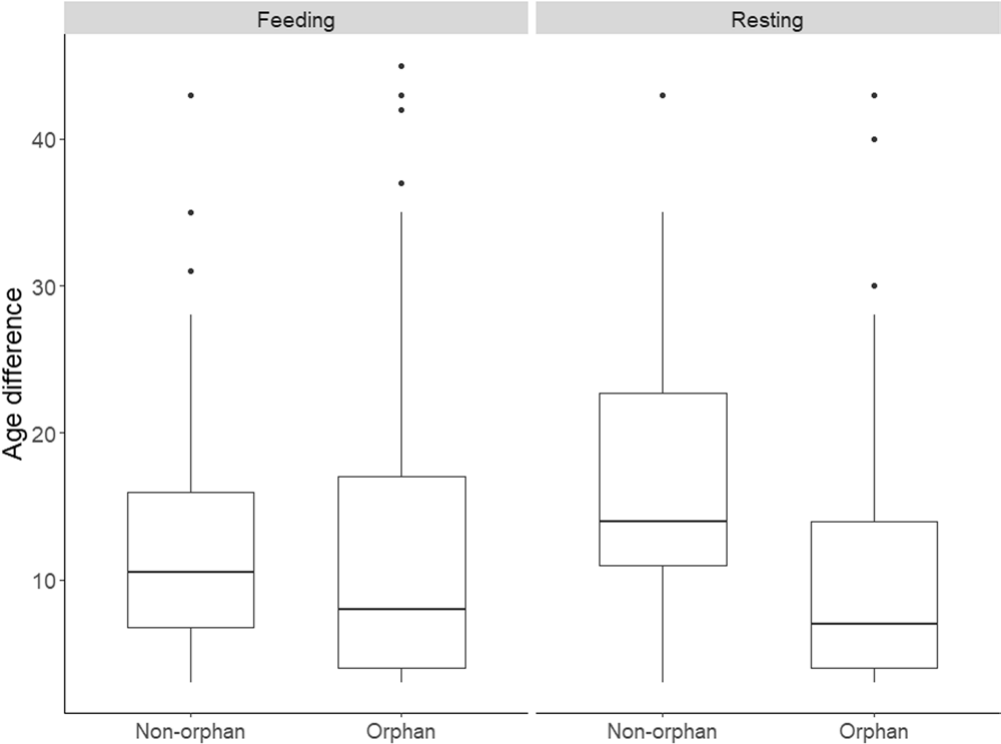
Orphan female adult social partners were generally younger than the female adult social partners of non-orphans, particularly while resting.

Orphans were more likely to interact with sisters and bulls while resting than were non-orphans (Tables 1 and 2). Resting non-orphans tended to affiliate more with calves than did orphans, but orphans tended to affiliate more with age mates than did non-orphans. Resting orphans tended not to affiliate with matriarchs, and resting non-orphans tended to affiliate with their mothers (Fig. 2). In contrast to feeding, resting orphans that lost their mothers at an earlier age affiliated more (Table 1).

## Discussion

The ability of orphans to adjust to their new circumstances presents a considerable challenge in socially dependent animals. Field studies of wild populations have demonstrated compensatory behavior of orphans in different taxa^11,13^, but the process of social integration following maternal loss is not well understood. Access to more experienced animals is thought to be one of the primary benefits of tight knit elephant social structure^19,25^, and whether or not orphans are able to compensate for lost relationships with adults may be consequential for their survival and reproduction. Our comparisons of social behavior in elephant orphans and non-orphans in this wild population offer insight into the drivers of elephant sociality and the challenges of orphaning in this society.

Our models indicated strong differences between orphan and non-orphan interaction partners, particularly while resting. Interactions while resting suggested that non-orphans affiliate more with young calves and their mothers, while orphans were primarily with younger individuals in the aggregation, notably sisters, age mates, and bulls. While feeding (behavior focused on widely distributed resources), both orphans and non-orphans exhibited more diverse social partners. Differences were apparent, however, most notably in avoidance of matriarchs by orphans and the lack of their mother as a social partner. The loss of their mother and apparently related changes in their social interactions indicate that orphans lack direct access to mature female elephants.

**Figure 3:**
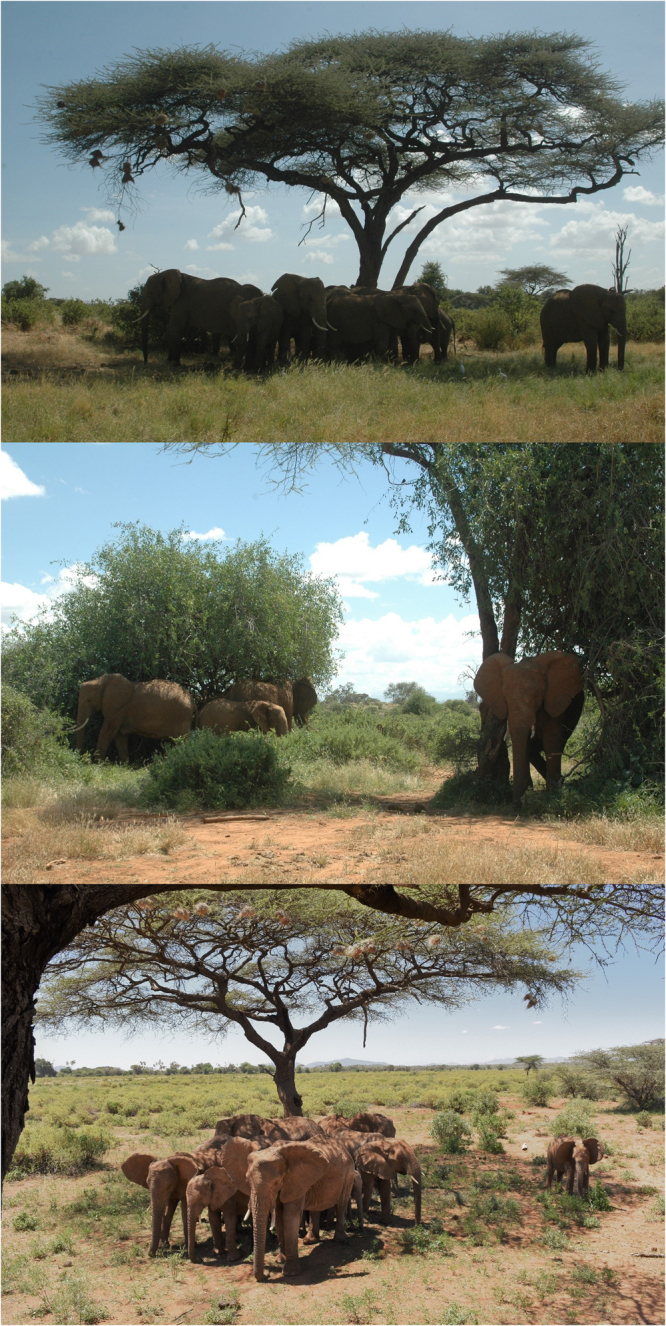
Orphans were often observed resting on the periphery of their social groups.

By assessing social contexts across activities with different resource competition, we gained insight to the implications of the observed loss of social access for orphans^5^. Reducing potential conflicts may be important to orphans when interacting in potentially competitive circumstances. We did not expect to find support for this during feeding when resources are more widespread, but orphans nonetheless exhibited lower than average interaction rates with matriarchs while feeding. Despite the widespread nature of foraging resources in this system, elephants exhibit clear preferences among resource patches^35^. The more limited social interaction between orphans and matriarchs while foraging indicates that access to high quality resource patches may vary depending on family history. Relatedly, the best positions during resting are typically secured by older, more dominant animals, when competition for limited shade is common^24,33^. Anecdotally, non-orphans tended to cluster at the core of family groups, whereas orphans were observed to occupy peripheral positions external to the group (Fig. 3), often in proximity to lower social status bulls, age mates, or sisters. These observations may also explain why non-orphans were more likely to interact with young calves while resting, which tend to be at the core of resting groups. Our results are consistent with a study of reindeer that found greater distances of orphans to adult females than non-orphans to adult females, especially in the context of spatially concentrated resources^5^. Lost opportunities to access needed resources may be an important factor for orphan fitness across taxa.

The differences apparent between orphans and non-orphans in access to older elephants suggest disadvantages to being orphaned. Similar to baboons^11,12^, elephant orphans increase affiliation with sisters in response to maternal loss (at least while resting), which may buffer them from the costs of orphaning. However, their compensatory bonding largely did not include adults (Fig. 2). Older female elephants confer known fitness advantages to their affiliates via ecological knowledge^36^, dominance^24^, social knowledge^19^, and calf survival^15,16^. Loss of access to mature animals, therefore, may lower the fitness of orphans via several mechanisms in this matriarchal society. Previous work has documented altered association patterns among female elephants in response to mortality, such that core social groups (defined by association patterns) are sometimes comprised of nonrelatives^13,32,37^. While this previous work demonstrates their behavioral flexibility in times of disruption, our current study provides finer scale detail on the social environments they experience. The behavioral flexibility and social processes of orphans are likely critical to population recovery in the face of the recent continental poaching crisis. Future work should investigate how decreased access to knowledge repositories of adult elephants and compensatory social behavior alter fitness trajectories for this keystone species.

## Methods

### Data Collection

We conducted near daily transects in Samburu and Buffalo Springs National Reserves in northern Kenya between May 2012 and April 2015 and between August and September 2017. Aggregations of elephants were encountered randomly along transects. For each aggregation, we recorded the individuals present and whether all breeding females were observed^38^. Association indices between pairs of elephants derived from high quality observations^39^ were used to define core social group membership, the strongest level of bonding in female elephant society^13,38^. While female elephants are usually found with their core social group, aggregations of elephants on any given day may consist of any combination of core groups and independent bulls, such that social context changes frequently^38^. Co-occurrence in aggregations was used to control for the availability of two elephants to interact in analyses (see below).

To determine the fine scale social patterns of elephants, we conducted focal follows (up to 30 minutes) of non-parous female elephants between six and 17 years old encountered along transects (Feeding: N_orphans_ = 28, N_median (IQR) minutes/orphan_ = 387.38 (96.75–540.19), N_median (IQR) follows/orphan_ = 20 (4.75–30), N_non-orphans_ = 19, N_median (IQR) minutes/non-orphan_ = 310.5 (119–386.13), N_median (IQR) follows/non-orphan_ = 19 (6–23.5); Resting: N_orphans_ = 30, N_median (IQR) minutes/orphan_ = 57 (30–86.88), N_median (IQR) follows/orphan_ = 3 (2–5), N_non-orphans_ = 18, N_median (IQR) minutes/non-orphan_ = 57.5 (34.13–86.19), N_median (IQR) follows/non-orphan_ = 2.5 (2–4.75); total sampling hours = 246.12 feeding and 49.14 resting; Supplementary Material Fig. 1). To control for behavioral autocorrelation, no more than one focal follow in a particular activity was completed on a given animal in a given sampling day, and follows were terminated before reaching 30 minutes if the animal went out of sight or switched activity. Competition for resources is expected to differ between feeding and resting because shade is a limited point resource whereas forage is more diffusely distributed. We therefore distinguished between focal follows collected during the two activities (so that a maximum of 60 minutes was collected on a focal animal in one day: 30 minutes resting and 30 minutes feeding). Sampling spanned both wet and dry seasons, and focal follows were only initiated if elephants appeared unperturbed by the research vehicle.

During focal follows we recorded any interactions with other elephants^33,40–42^. For this study on social bonding, we restricted analysis to affiliative interactions indicative of bonding including bodily contact, trunk touching, greeting, allomothering, play behavior, and reaching a trunk to another elephant’s mouth^40^. We conducted research with permission from the Kenya Wildlife Service, Samburu and Isiolo governments, and Colorado State University (IACUC 12–3414 A), and all methods adhere to relevant guidelines and regulations. Data used in this study are included as Supplementary Material.

### Data Analysis

In order to determine general differences between orphans and non-orphans, we conducted Kruskal-Wallis rank sum tests to assess the number of social partners per time followed and the age differences between social partners and focal animals. We conducted an additional Kruskal-Wallis rank sum test on age differences including only female partners older than focal animals to more directly assess affiliation with older individuals in female society.

To understand how orphans and non-orphans differ in the categories of their social partners, we constructed four hierarchical Bayesian negative binomial regression models with uninformative priors predicting affiliative interactions with social partners during different activities (orphans feeding, non-orphans feeding, orphans resting, non-orphans resting). We used the following process model:

1 $$\mathrm{ln}({\lambda }_{i,j})={\alpha }_{j}+{\boldsymbol{\beta }}{{\boldsymbol{x}}}_{i,j}+\,\mathrm{ln}({\gamma }_{i,j}),$$

where *λ*_*i,j*_ is the expected interaction count for elephant *j* with social partner *i* (across all focal follows of elephant *j* during which social partner *i* was observed in the same aggregation), *α*_*j*_ is the random intercept for elephant *j*, ***β*** represents the vector of fixed effects coefficients associated with covariates ***x***, and ln(*γ*_*i,j*_) is an offset controlling for sampling effort (the total number of focal minutes for elephant *j* during which social partner *i* was observed in the aggregation and therefore available to interact). The conditional probability was defined as:

2 $$({y}_{i,j}|{\boldsymbol{\beta }},{\alpha }_{j},{\mu }_{\alpha },{\tau }_{\alpha },r)\sim negbinom({p}_{i,j},r)$$

3 $${\boldsymbol{\beta }}\, \sim normal(0,0.1)$$

4 $${\alpha }_{j}\, \sim normal({\mu }_{\alpha },{\tau }_{\alpha })$$

5 $${\mu }_{\alpha }\, \sim normal(0,0.1)$$

6 $${\tau }_{\alpha } \sim uniform(0.001,100)$$

7 $$r\, \sim uniform(0,100)$$

where *µ*_*α*_ and *τ*_*α*_ are the mean and precision of *α*_*j*_, respectively, *r* is the dispersion parameter, and *p*_*i,j*_ is the probability that an interaction occurs.

Predictor variables included social partner categories and control variables that might affect interactions using information available from the long-term records of the population (Table 3). No covariates included in a model were correlated above r = |0.7|. We fit models using JAGS^43^ and the *rjags* package in R^44,45^ with Markov-Chain Monte Carlo by running three parallel chains of 100,000 iterations each, and discarded the first 10% of iterations as burn-in after assessing convergence indicated by Gelman-Rubin diagnostic values < 1.1^46^.

**Table 3:** Model covariates, definitions, and their inclusion in orphan or non-orphan models.

Covariate	Definition	Orphan models	Non-orphan models
Age	Average age in years across focal follows	X	X
Age orphaned	Focal animal age at which mother died	X	
Age mate	+/− 2 years of focal animal’s age	X	X
Aunt	Focal animal’s adult maternal aunt	X	X
Bull	Male dispersed from his natal group	X	X
Calf	≤6 months old at any point during the study	X	X
Matriarch	Oldest member of a core social unit that is not the focal animal’s mother	X	X
Mother	Focal animal’s mother		X
Sister	Focal animal’s maternal sister	X	X

While grandmothers are important social partners in undisrupted elephant society, there were too few grandmothers alive during our study to include this partner category.

## Electronic supplementary material

Supplementary Material

Dataset feeding

Dataset resting
