# Supplementary material for: Orphaned female elephant social bonds reflect lack of access to mature adults
Source: Sci Rep. 2017 Oct 31;7:14408. doi: 10.1038/s41598-017-14712-2 (PMC5663962; doi:10.1038/s41598-017-14712-2)
Supplement: Supplementary file 1 — Supplementary Material [file 41598_2017_14712_MOESM1_ESM.pdf]

Orphaned female elephant social bonds reflect lack of access to mature adults

Shifra Z. Goldenberg[1,2*] and George Wittemyer[1,2]

[1]Department of Fish, Wildlife, and Conservation Biology, Fort Collins, CO 80523, USA

[2]Save the Elephants, Nairobi 00200, Kenya

*Correspondence: shifra.z.goldenberg@gmail.com

Supplementary Material

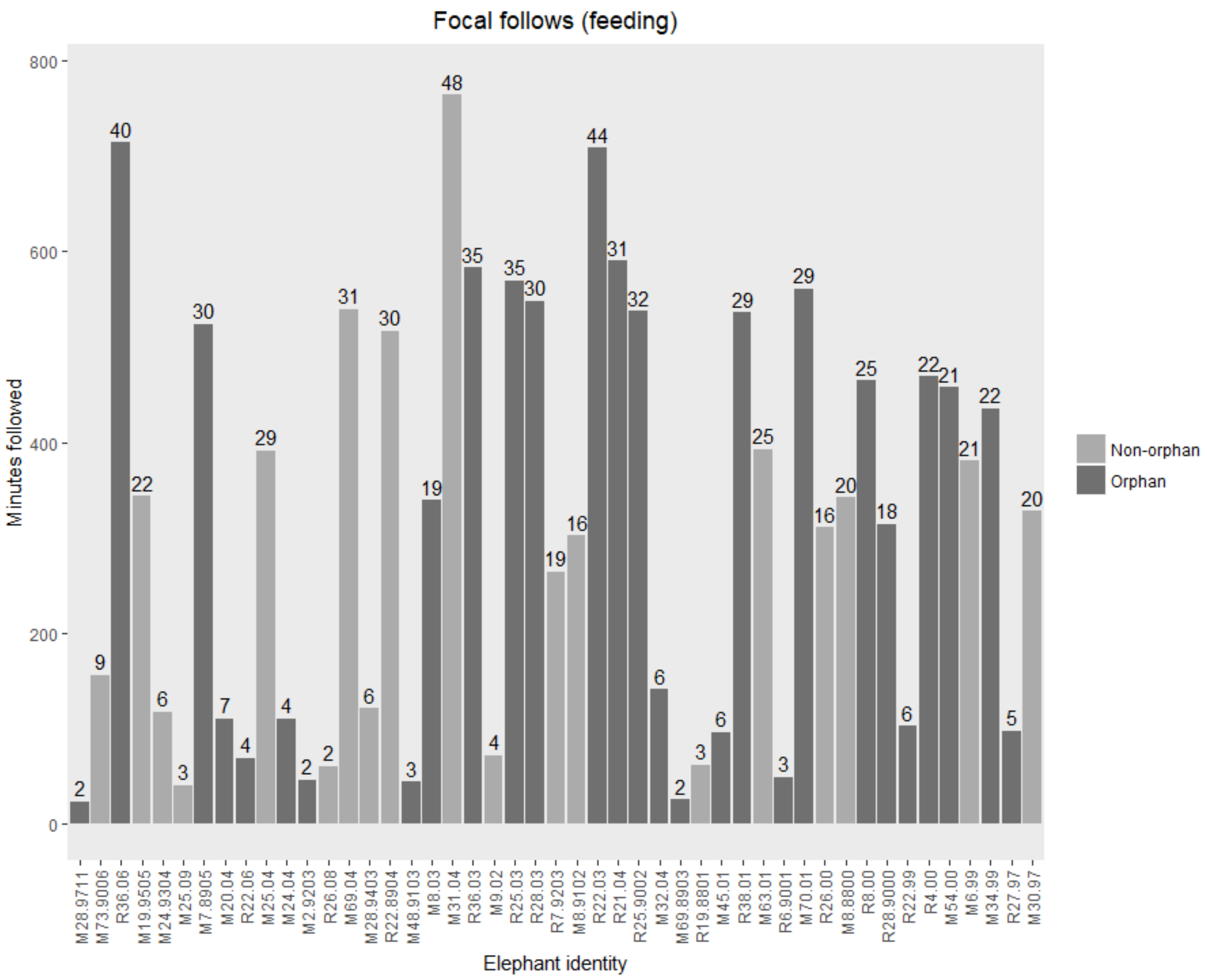

Focal follows (feeding)
Minutes followed
Elephant identity
Non-orphan
Orphan
M28.9711
M73.9006
R36.06
M19.9505
M24.9304
M25.09
M7.8905
M20.04
R22.06
M25.04
M24.04
M2.9203
R26.08
M69.04
M28.9403
R22.8904
M48.9103
M8.03
M31.04
R36.03
M9.02
R25.03
R28.03
R7.9203
M8.9102
R22.03
R21.04
R25.9002
M32.04
M69.8903
R19.8801
M45.01
R38.01
M63.01
R6.9001
M70.01
R26.00
M8.8800
R8.00
R28.9000
R22.99
R4.00
M54.00
M6.99
M34.99
R27.97
M30.97

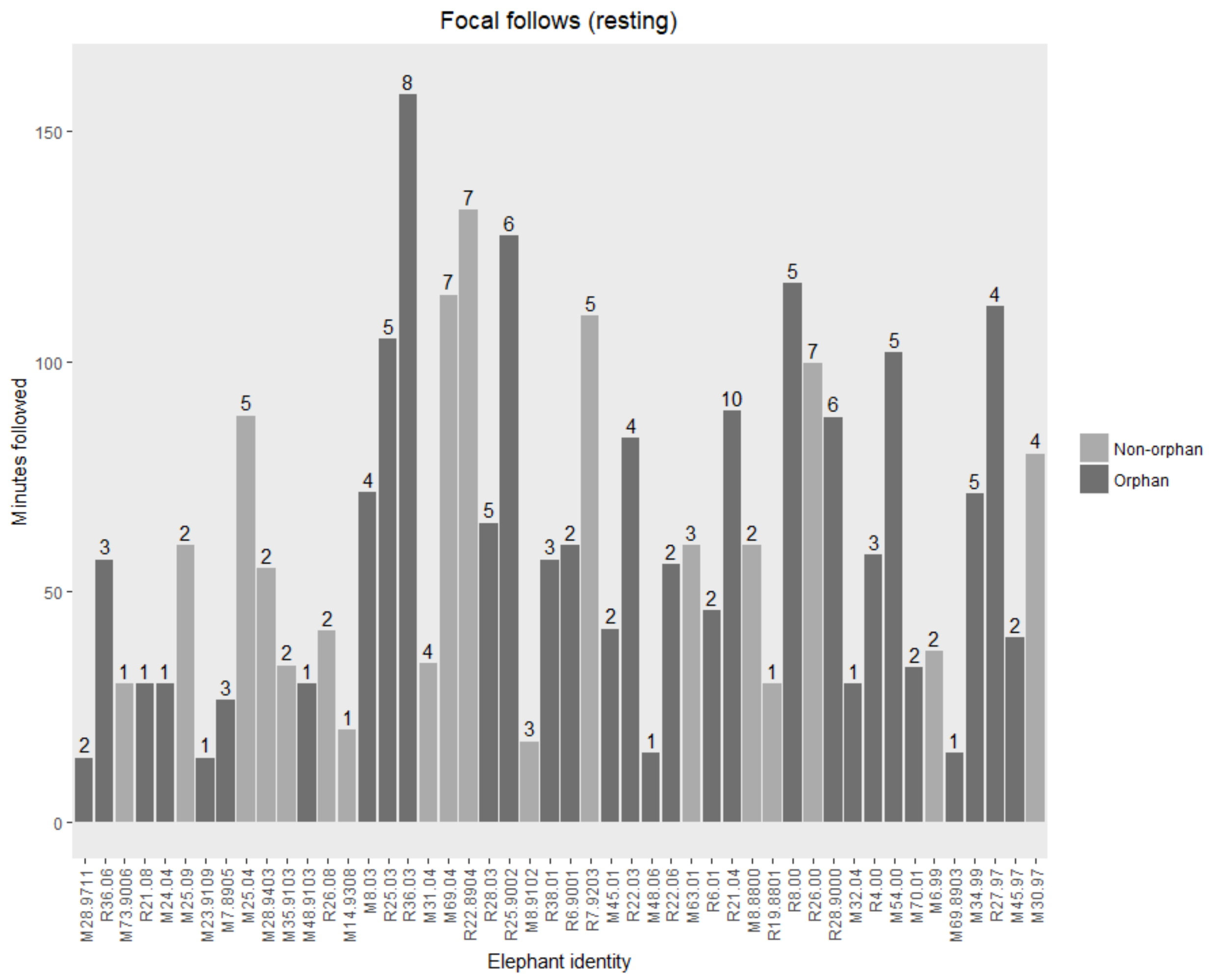

Figure 1. Distribution of focal follow data for feeding and resting animals. Elephants are ordered from left to right by the youngest mean age during her focal follows (Feeding and resting: 6 years) to the oldest (Feeding: 15.95 years, Resting: 15.75 years). Numbers above bars indicate the number of discrete focal follows (i.e., discrete sampling days) represented within each bar.

Additional analyses:

To ensure that variables that could only be included for either orphans or non-orphans (mother, age orphaned) did not preclude comparison between models, we ran additional analyses excluding these variables, finding consistent results across analyses.

Two covariates of interest were only available for either orphans or non-orphans (mother, age orphaned). To ensure that inclusion of these covariates did not preclude comparison between orphans and non-orphans, we ran the same analyses excluding these variables. Results (Table 1) were similar to those of the full models, which are included in the main text.

Table 1. Median (95% credible interval) posterior distribution estimates of regression coefficients

	**Age**	**Age mate**	**Aunt**	**Bull**	**Calf**	**Matriarch**	**Sister**
**Orphans**	-0.342	0.399	-0.583	0.703	0.903	-0.490	-0.408
**(feeding)**	(-0.557--0.139)	(-0.004-0.803)	(-1.633-0.579)	(0.289-1.114)	(0.558-1.244)	(-1.102-0.139)	(-1.011-0.229)
**Non-orphans**	0.038	0.611	-0.830	0.831	1.169	-0.144	0.250
**(feeding)**	(-0.177-0.226)	(0.146-1.097)	(-1.885-0.268)	(0.313-1.364)	(0.789-1.552)	(-1.202-0.956)	(-0.311-0.853)
**Orphans**	-0.205	0.493	-0.094	1.138	0.058	-0.310	1.010
**(resting)**	(-0.506-0.110)	(-0.074-1.083)	(-1.328-1.377)	(0.329-1.994)	(-0.508-0.643)	(-1.086-0.544)	(0.369-1.690)
**Non-orphans**	-0.004	0.215	0.938	0.252	0.166	0.088	-0.499
**(resting)**	(-0.229-0.234)	(-0.408-0.885)	(-0.697-3.073)	(-0.516-1.117)	(-0.405-0.764)	(-1.331-1.897)	(-1.321-0.470)
